# Targeting PSMD14 inhibits melanoma growth through SMAD3 stabilization

**DOI:** 10.1038/s41598-020-76373-y

**Published:** 2020-11-05

**Authors:** Satoru Yokoyama, Yusuke Iwakami, Zhao Hang, Ryoei Kin, Yue Zhou, Yutaka Yasuta, Atsushi Takahashi, Yoshihiro Hayakawa, Hiroaki Sakurai

**Affiliations:** 1 Department of Cancer Cell Biology, Faculty of Pharmaceutical Sciences, University of Toyama, 2630 Sugitani, Toyama, 930-0194 Japan; 2 Division of Pathogenic Biochemistry, Institute of Natural Medicine, University of Toyama, 2630 Sugitani, Toyama, 930-0194 Japan

**Keywords:** Skin cancer, Cell growth, Deubiquitylating enzymes

## Abstract

Although melanoma therapy is improved by novel molecular targeted reagents, including vemurafenib, aberrant proliferation and early metastasis remain obstacles for melanoma; therefore, novel target molecules for melanoma need to be identified. In this study, we focused on deubiquitinating enzymes, which regulate protein stability through ubiquitin–proteasome systems, and identified 26S proteasome non-ATPase regulatory subunit 14 (PSMD14) as a molecule related to melanoma growth using siRNA library screening. Similar to a previous report, PSMD14 knockdown strongly induced p21 expression and inhibited RB phosphorylation in melanoma. After in silico analysis, TGF-β signaling was identified as a negatively correlated gene set with PSMD14 expression. Although TGF-β signaling is also related to the invasive phenotype of melanoma, PSMD14 knockdown suppressed melanoma migration and reduced SLUG expression, suggesting that targeting PSMD14 suppresses both growth and migration. Furthermore, SMAD3 expression increased in nucleus and SMAD3 degradation was delayed after PSMD14 knockdown. Thus, our present study suggests that targeting PSMD14 can inhibit melanoma growth and migration through either SMAD3 accumulation or SLUG reduction, respectively.

## Introduction

Aberrant proliferation and metastatic ability are the hallmarks of cancer malignancy, including melanoma. Proliferation is highly accelerated by the mutation of oncogenes BRAF and NRAS^1^, and targeting BRAF^2^ and MEK1/2^3^ has progressively improved melanoma therapy in the past few decades; however, its benefit are limited and almost all patients eventually relapse^2–5^. Thus, the identification of novel therapeutic target molecules for melanoma growth is urgently needed.

The ubiquitin–proteasome system is one of the protein degradation pathways, and is tightly regulated by two types of enzymes, ubiquitin ligation-related enzymes (E1 to E3) and deubiquitinating enzymes (DUBs). The DUB family contains more than 100 proteins, which remove ubiquitin from numerous substrates^6^. The biological functions of DUBs have been clarified over the past decade and some DUBs are related to cancer pathogenesis like cancer growth, cancer metastasis, and immune evasion^7–9^. The aberrant cell cycle in melanoma is caused by disturbed ubiquitination and degradation of cyclins or cyclin-dependent kinase inhibitors, such as p21 (*CDKN1A*) and p27 (*CDKN1B*)^10^. In addition, the metastatic ability of melanoma is regulated by post-translational modification of SLUG transcription factor^9^. As such, we focused on DUBs as molecular targets for melanoma.

In this study, we identified proteasome 26S proteasome non-ATPase regulatory subunit 14 (PSMD14) as a regulator of melanoma growth using a siRNA library for DUBs. Using gene set enrichment analysis of the negatively correlated genes with *PSMD14* in melanoma, TGF-β signaling was identified as an enriched gene sets. Although many reports suggested the anti-proliferative and pro-metastatic effects of TGF-β signaling in melanoma^11–13^, PSMD14 knockdown suppressed both proliferation and migration. In addition, we noted the stabilization and induction of SMAD3 expression after PSMD14 knockdown. On the other hand, SLUG, an epithelial-to-mesenchymal transition-related transcription factor, was significantly reduced after PSMD14 knockdown. Lastly, the effects of SMAD3 induction on growth suppression were clarified by the knockdown of SMAD3. Our study suggests that PSMD14 is a good molecular target for both proliferation and metastasis.

## Materials and methods

### Cell culture

UACC257, M14, A2058, Malme-3M, SK-MEL-28, and MeWo cell lines were purchased from ATCC, and cultured in RPMI1640 supplemented with 2 mM l-glutamine, 10% fetal bovine serum, 100 units/mL of penicillin, and 100 μg/mL of streptomycin. The cells were maintained at 37 °C in a humidified atmosphere of 5% CO_2_.

For small interfering RNA (siRNA) screening, 12.5 nM Dharmacon siGENOME SMARTpool siRNA Library (Human Deubiquitinating Enzymes) and siGENOME Non-targeting siRNA Pool #2 (Thermo Fisher Scientific, Rockford, IL, USA) were reverse-transfected to UACC257 and M14 cells using Lipofectamine RNAiMAX reagent (Thermo Fisher Scientific). Other siRNAs were purchased from Thermo Fisher Scientific. For siRNA knockdown experiments, siRNA for PSMD14 (siPSMD14) (s19919 and s19920), siRNA for UBL5 (siUBL5) (s224521, s224522), siRNA for BAP1 (siBAP1) (s15821, s15822), siRNA for SMAD2 (s8397), siRNA for SMAD3 (s8402), or negative control #1 (siCNTL) was transfected at a final concentration of 12.5 nM into UACC257 or M14 cells using Lipofectamine RNAiMAX reagent. Transfected cells were subjected to CellTiter-Glo luminescent cell viability assay, Western blotting, and qRT-PCR after 96 h.

### Cell growth assay

Melanoma cells were seeded with reverse-transfection of siRNA library or individual siRNA. After a 96-h incubation, WST-1 solution (Dojindo, Kumamoto, Japan) was added according to the manufacturer’s instructions. The absorbance at 450 nm was measured by a Microplate reader.

### Western blotting

Western blotting was performed as described previously^14^. Briefly, whole cell lysates were collected in whole cell lysis buffer (20 mM HEPES pH 7.6, 0.5 M NaCl, 1.5 mM MgCl_2_, 1 mM EDTA, 0.1% Triton X-100, and protease inhibitors (20 mM β-glycero-phosphate, 1 mM sodium orthovanadate, 1 mM phenyl-methylsulfonyl fluoride, 1 mM dithiothreitol, 10 mg/mL aprotinin, and 10 mg/mL leupeptin)). For nuclear/cytoplasmic protein preparation, cells were lysed in lysis buffer C (20 mM HEPES pH 7.6, 0.04 M NaCl, 1.5 mM MgCl_2_, 1 mM EDTA, 0.1% Triton X-100, and protease inhibitors). After centrifugation, the supernatants were collected as cytoplasmic extracts. The precipitates were again lysed in whole cell lysis buffer and collected as nuclear extracts. Equal amounts of protein were resolved by electrophoresis on 7.5% or 10% acrylamide gels and transferred to polyvinylidene difluoride (PVDF) membranes. The primary antibodies used were phospho-RB (S807/811) (9308), RB (9313), p21 (2947), p27 (2552), PSMD14 (4197), SMAD2/3 (5678), and SMAD3 (9523) from Cell Signaling Technology (Beverly, MA, USA), α-tubulin (T5168) from Sigma-Aldrich (St. Louis, MO, USA), and Lamin B (sc-6216) from Santa Cruz Biotechnology. The antibodies were detected using horseradish peroxidase-conjugated anti-rabbit (P0448, DAKO, Glostrup, Denmark), anti-mouse (P0260, DAKO), and anti-goat IgG (P0449, DAKO), and visualized by the ECL system (GE healthcare Bioscience, Piscataway, NJ, USA). The band intensities were measured by ImageJ and normalized to that of each control lane. Uncropped scans of the blots are supplied in Supplemental Fig. S3.

### Real time PCR

Total RNA was prepared using the RNeasy Plus Mini kit (Qiagen, Hilden, Germany) and subjected to real-time PCR on an ABI Prism 7300 sequence detection system (Life Technologies Corporation, Carlsbad, CA USA). The expression level of each mRNA was normalized to that of β*-actin* mRNA. The primers used were: 5′-AGT CAG TTC CTT GTG GAG CC-3′ (sense) and 5′-CAT GGG TTC TGA CGG ACA T-3′ (antisense) for *p21* mRNA, 5′-TGC AAC CGA CGA TTC TTC TAC TCA A-3′ (sense) and 5′-CAA GCA GTG ATG TAT CTG ATA AAC AAG GA-3′ (antisense) for *p27* mRNA, 5′-GGA GGA GGT ATG CCT GGA CT-3′ (sense) and 5′-TTA ACA GTG CCA GGG AAG AGA-3′ (antisense) for *PSMD14* mRNA, 5′-CAC GCT AGG AAA ACA GCC TC-3′ (sense) and 5′-TCG GAA GAG GAA GGA ACA AA-3′ (antisense) for *SMAD2* mRNA, 5′-TCA ACA CCA AGT GCA TCA CC-3′ (sense) and 5′-CGG CAG TAG ATG ACA TGA GG-3′ (antisense) for *SMAD3* mRNA, and 5′-GCA CAG AGC CTC GCC TT-3′ (sense) and 5′-GTT GTC GAC GAC GAG CG-3′ (antisense) for *β-actin* mRNA.

### Gene set enrichment analysis

Gene expression data of melanoma cell lines were downloaded from Cancer Cell line Encyclopedia (https://portals.broadinstitute.org/ccle)^15^. After picking up the top 1% genes negatively correlated with PSMD14 expression, gene set enrichment analysis was performed using the molecular signatures database (https://www.gsea-msigdb.org/gsea/msigdb/index.jsp)^16,17^.

### Protein chase assay

Cells were reverse-transfected with siRNAs for 96 h and then were treated with the protein synthesis inhibitor cycloheximide (Cayman chemical, Ann Arbor, MI, USA). After each time point, whole cell lysates were collected and subjected to Western blotting.

### Migration assay

The migration assay was performed as described previously^18^. Briefly, membrane filters (Whatman, Maidstone, UK) were attached to Transwell chambers (Costar, Cambridge, MA, USA) and the lower surface was pre-coated with 1.25 μg of laminin (Iwaki, Tokyo, Japan). Ninty-six hours after transfecting siPSMD14, transfected cells (5 × 10^4^ cells/200 μL of RPMI 1640 medium with 0.1% bovine serum albumin) were added to the upper compartment of the chamber. After incubation for 9 h for UACC257 cells, 6 h for M14 cells, 14 h for A2058, and 4.5 h for SK-MEL-28 cells, the migrated cells were stained by hematoxylin and eosin, and counted manually under a microscope at × 50 magnification.

### Chromatin immunoprecipitation assay (ChIP assay)

ChIP assays were performed as described previously^14^. The antibodies used were anti-polymerase II serine2 phosphorylation antibody (Abcam, MA, USA) and anti-SMAD3 antibody. Primers used were; 5′-CAG CTG AGG TGT GAG CAG-3′ (sense) and 5′-CCT CTG AGT GCC TCG GT-3′ (antisense) for *p21* gene region, 5′-ACT TGT CCC TAG GAA AAT CC-3′ (sense) and 5′-GAA AAC GGA GAG TGA GTT TG-3′ (antisense) for SMAD binding site on *p21* promoter region. ChIP samples were subjected to real-time PCR on an ABI Prism 7300 sequence detection system.

### Statistical analysis

Significance was assessed using Graphpad Prism software (GraphPad Software, Inc, San Diego, CA). More than three means were composed using one-way ANOVA with the Bonferroni correction. P < 0.05 was considered significant.

## Results

### PSMD14 is related to melanoma growth

**Figure 1:**
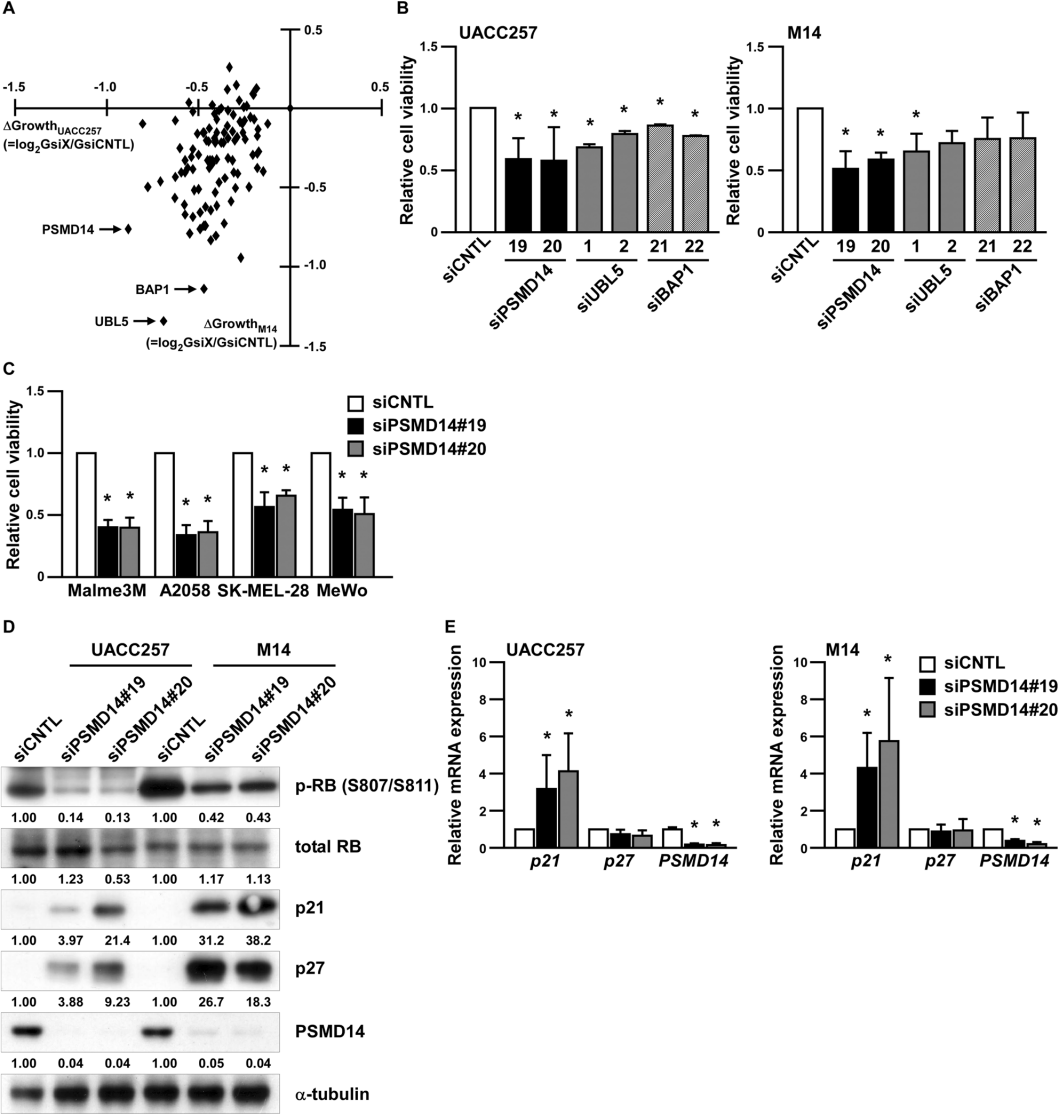
PSMD14 is related to melanoma growth. (**A**) UACC257 and M14 cells were transfected with a siRNA library against deubiquitinating enzymes for 96 h and subjected to the cell growth assay using WST-1. (**B**) UACC257 and M14 cells were transfected with siRNA against negative control (siCNTL), PSMD14 (siPSMD14), UBL5 (siUBL5), or BAP1 (siBAP1) for 96 h, and subjected to the cell growth assay using WST-1. (**C**) Human melanoma cell lines were transfected with each indicated siRNA for 96 h and subjected to the cell growth assay using WST-1. Data are shown as the mean ± SD of at least three independent experiments. *P < 0.01 vs siCNTL-transfected cells by one-way ANOVA followed by the Bonferroni post-hoc test. (**D**,**E**) UACC257 and M14 cells were transfected with each indicated siRNA for 96 h, and subjected to Western blotting (**D**) and real-time RT-PCR (**E**). *P < 0.01 vs siCNTL-transfected cells by one-way ANOVA followed by the Bonferroni post hoc test.

To identify potential DUBs responsible for melanoma growth, we knocked-down 97 individual human DUBs by pooled siRNA (four siRNAs per gene) in UACC257 and M14 human melanoma cells (Fig. 1A). After screening, we identified 3 genes, 26S proteasome non-ATPase regulatory subunit 14 (*PSMD14*), Ubiquitin-like protein 5 (*UBL5*), and BRCA1 Associated Protein 1 (*BAP1*), as DUBs related to melanoma growth. Among them, PSMD14 knockdown reduced melanoma growth using two independent siRNAs, instead of pooled siRNA, in UACC257 and M14 cells (Fig. 1B). Moreover, PSMD14 knockdown suppressed the growth of Malme-3M, A2058, SK-MEL-28, and MeWo cell lines (Fig. 1C). Similar to a previous report^19^, p21, whose gene name is CDKN1A, induction and phosphorylation of RB were detected after PSMD14 knockdown (Fig. 1D). We then further investigated whether PSMD14 regulates mRNA expression levels of *p21* in melanoma cells. As shown in Fig. 1E, PSMD14 knockdown significantly induced the expression of *p21* mRNA in melanoma cells, but not the expression of *p27* mRNA, whose gene name is CDKN1B. This suggests that PSMD14 transcriptionally regulates *p21* expression and post-transcriptionally regulates *p27* expression in melanoma, thereby affecting cell growth.

### PSMD14 regulates SMAD3 stability in melanoma

**Figure 2:**
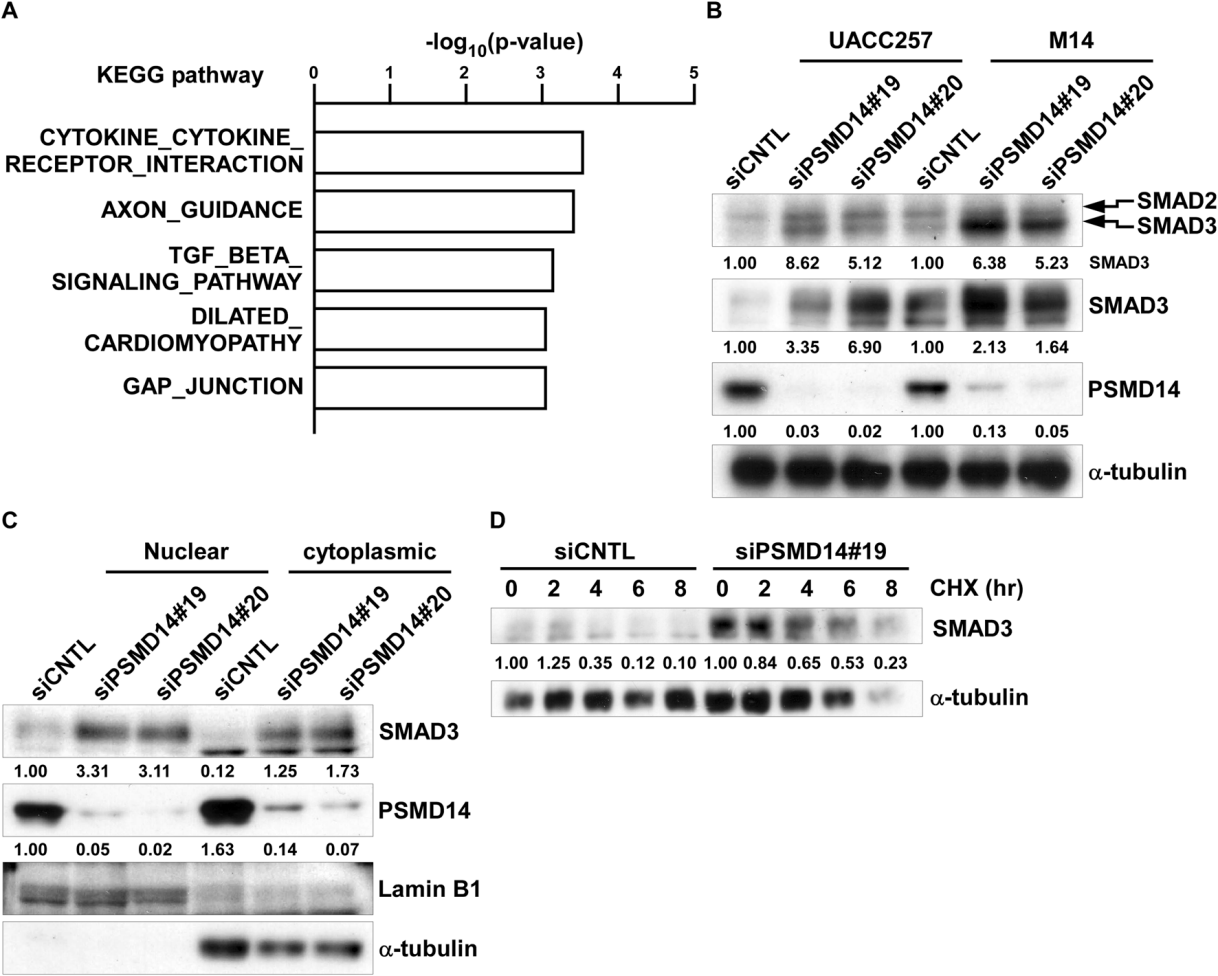
PSMD14 regulates SMAD3 stability in melanoma. (**A**) Top 1% of genes negatively correlated with PSMD14 were extracted from melanoma data in the Cancer Cell Line Encyclopedia. The extracted top 1% genes were analyzed by the gene set enrichment analysis using the Molecular Signatures Database. KEGG pathways significantly enriched are shown. (**B**) UACC257 and M14 cells were transfected with PSMD14 siRNA for 96 h. The whole cell lysates were subjected to Western blotting. (**C**) Nuclear or cytoplasmic protein was subjected to Western blotting. Other conditions were as in (**B**). (**D**) UACC257 cells were transfected with PSMD14 siRNA for 96 h. The transfected cells were treated with 50 μg/mL of cycloheximide for the indicated times and subjected to Western blotting. The band intensities were measured by ImageJ, normalized to that at 0 h for each cell lines, and shown below each panel.

As PSMD14 is a subunit of 19S regulatory particles of 26S proteasomes and is related to protein degradation^20^, we examined related signaling pathways in PSMD14 knockdown in melanoma using the Molecular Signatures Database (https://www.gsea-msigdb.org/gsea/msigdb/index.jsp)^16,17^. Using melanoma data sets from Cancer Cell Line Encyclopedia^15^, the gene set related to TGF-β signaling was significantly enriched among the top 1% genes negatively correlated with PSMD14 expression (Fig. 2A). As *p21* transcription is regulated through SMAD2/3^21^, which are downstream molecules of TGF-β signaling, we next examined the expression of SMAD3 at the protein levels after PSMD14 knockdown. As shown in Fig. 2B, SMAD3 protein expression was strongly induced, although *SMAD3* mRNA was slightly induced after PSMD14 knockdown (data not shown). Furthermore, increased SMAD3 expression in the nucleus was observed in UACC257 and M14 cells (Fig. 2C, Supplemental Fig. S1), supporting the translocation of SMAD3 to the nucleus. In order to examine the effects of PSMD14 knockdown on SMAD3 degradation, UACC257 and M14 cells were treated with the protein synthesis inhibitor cycloheximide. As shown in Fig. 2D and Supplemental Fig. S1, SMAD3 stability was prolonged after PSMD14 knockdown. This suggests that SMAD3 degradation is regulated through PSMD14.

### Targeting PSMD14 inhibits melanoma migration and SLUG expression

**Figure 3:**
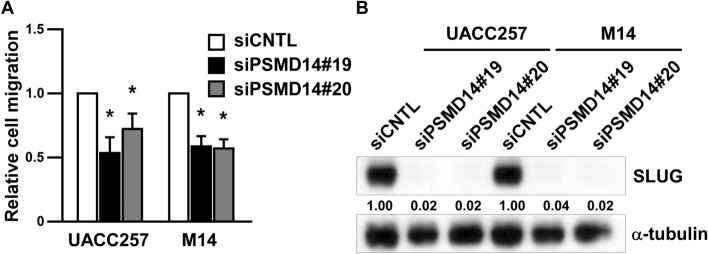
Targeting PSMD14 inhibits melanoma migration and SLUG expression. (**A**) UACC257 cells were transfected with PSMD14 siRNA for 96 h and then subjected to the migration assay. *P < 0.01 vs siCNTL-transfected cells by one-way ANOVA followed by the Bonferroni post hoc test. (**B**) UACC257 cells were transfected with PSMD14 siRNA for 96 h. The whole cell lysates were subjected to the Western blotting.

As TGF-β signaling is related to metastatic ability of cancer^22,23^, we investigated whether targeting PSMD14 in melanoma affects metastatic ability such as migration. PSMD14 knockdown slightly but significantly inhibited melanoma migration (Fig. 3A, Supplemental Fig. S2). Although SMAD3 is induced after PSMD14 knockdown (Fig. 2B), we detected the reduced expression of SLUG (Fig. 3B, Supplemental Fig. S2), one of the transcription factors responsible for melanoma metastasis^24^, suggesting that targeting PSMD14 inhibits melanoma migration through SLUG reduction.

### PSMD14 regulates melanoma growth through the SMAD3-p21 axis

**Figure 4:**
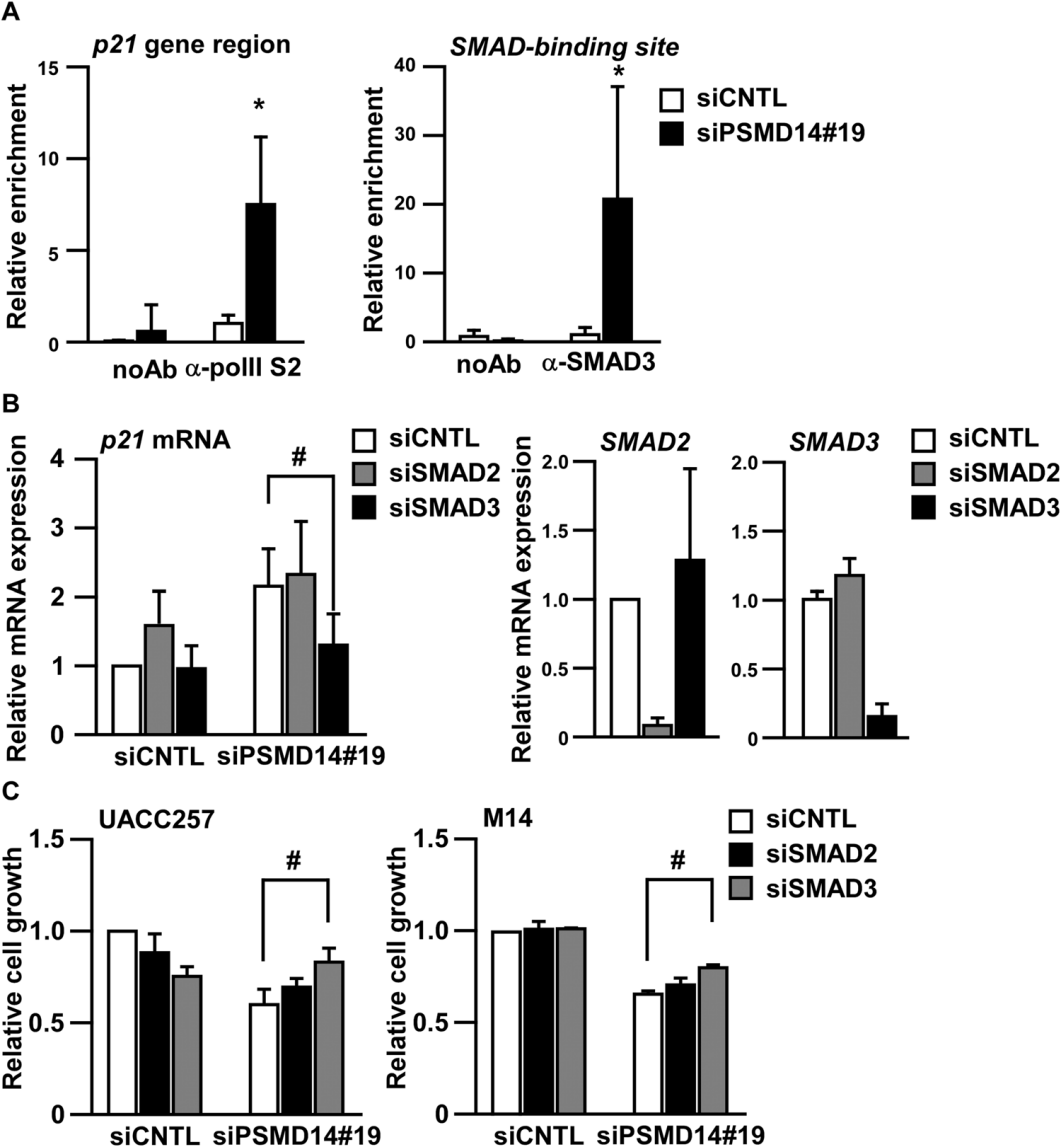
PSMD14 regulates melanoma growth through the SMAD3-p21 axis. (**A**) UACC257 cells were transfected with the indicated siRNAs for 96 h. The chromatin immunoprecipitation assay was performed using the antibody against polymerase II phosphorylated serine 2 site (α-pol II S2) or SMAD3 (α-SMAD3). Immunoprecipitated DNA was quantified by real-time PCR using primers specific to the gene region of *p21* or the SMAD-binding site on the p21 promoter. Results are normalized to the precipitated DNA using α-pol II S2 or α-SMAD3 in siCNTL-transfected cells. Data are presented as the mean ± SD of three independent experiments. *P < 0.01 vs. α-pol II S2-precipitated DNA or α-SMAD3 in siCNTL-transfected cells by two-way ANOVA followed by the Bonferroni post test. (**B**) UACC257 cells were transfected with the indicated siRNAs for 96 h. Relative mRNA expression was measured by quantitative real-time RT-PCR. (**C**) After 96 h, the transfected cells were subjected to the cell growth assay using WST-1. ^#^P < 0.01 vs siCNTL/siPSMD14#19-transfected cells by one-way ANOVA followed by the Bonferroni post hoc test.

Next to assess the relationship between SMAD3 and p21 in the context of targeting PSMD14, chromatin immunoprecipitation assay was performed using antibodies against RNA polymerase II (Ser2 phosphorylation) (pol-II S2) or SMAD3. As shown in Fig. 4A, the *p21* gene region with pol-II S2 was enriched after PSMD14 knockdown and the SMAD-binding site with SMAD3 was significantly enriched after PSMD14 knockdown. This suggests that both *p21* transcription and SMAD3 binding to the *p21* promoter region were induced after PSMD14 knockdown.

To clarify the functional significance of SMAD3 in the siPSMD14-mediated *p21* mRNA induction, we investigated the effects of SMAD2 or SMAD3 knockdown on *p21* mRNA expression. Although PSMD14 knockdown induced *p21* mRNA, its induction was significantly suppressed by SMAD3 knockdown, but not by SMAD2 knockdown (Fig. 4B) through the specific knockdown of either *SMAD2* mRNA or *SMAD3* mRNA. Consistent with *p21* mRNA expression, SMAD3 knockdown restored the siPSMD14-mediated growth suppression in UACC257 and M14 cells (Fig. 4C). This suggests that PSMD14 regulates melanoma growth through the SMAD3-p21 axis.

## Discussion

PSMD14, a DUB, is a subunit of 19S regulatory particles of 26S proteasomes, which deubiquitinate substrates and leads them to proteasomal degradation by 20S core particles^25,26^. As PSMD14 was reported to regulate melanoma growth^19^ and higher PSMD14 expression was correlated with a poor prognosis of melanoma (PrognoScan)^27^, PSMD14 may be a good molecular target for melanoma; however, the molecular mechanism underlying the suppression of melanoma growth by targeting PSMD14 is unclear. In this study, we identified SMAD3, but not SMAD2, as one of the key molecules related to p21 induction after PSMD14 knockdown (Fig. 4B). As both SMAD2 and SMAD3 are TGF-β downstream effectors with 92% amino acid sequence similarity, it may be difficult to distinguish the function of SMAD3 from that of SMAD2. Considering the general role of SMAD3, but not SMAD2, in DNA binding^28–30^ and its binding to the *p21* promoter region after PSMD14 knockdown (Fig. 4B), it is rational that growth was restored more by SMAD3 knockdown than by SMAD2 knockdown after PSMD14 knockdown (Fig. 4C). Although main focus of this study was p21 induction by PSMD14, p27 protein was also significantly increased after PSMD14 knockdown (Fig. 1D). In contrast to *p21* mRNA induction, *p27* mRNA was not increased after PSMD14 knockdown (Fig. 1E). As p27 degradation is regulated through SKP2^31^, PSMD14 knockdown may suppress SKP2 expression or impair the proteasomal degradation of p27 protein. As SKP2 reduction was observed after PSMD14 knockdown (data not shown), the inhibition of proteasome activity by PSMD14 knockdown and the inhibition of p27 degradation through SKP2 reduction may cause the accumulation of p27 protein in melanoma. Thus, PSMD14 may be an attractive target for melanoma through transcriptional regulation of p21 and post-transcriptional regulation of p27.

Recent reports identified intra-tumoral heterogeneity in melanoma biopsies and in short-term cultured melanoma cells, defining the two populations as proliferative phenotype or invasive phenotype^22,32^. This intra-tumoral heterogeneity is strongly related to intrinsic/acquired drug-resistance^33,34^ and melanoma metastasis^35^. As TGF-β signaling is known as a marker for the invasive phenotype^22,23^, we assessed if PSMD14 knockdown induces melanoma migration; however, melanoma migration was inhibited after PSMD14 knockdown (Fig. 3A) together with SLUG reduction, suggesting that the inhibition of migration is independent of SMAD3 induction in melanoma. We also confirmed that *SLUG* mRNA levels were not affected by PSMD14 knockdown (data not shown). As SLUG protein reduction was observed after PSMD14 knockdown (Fig. 3B), SLUG protein may be degraded through lysosomal degradation or 20S proteasomal degradation independent of PSMD14, or the translation of SLUG mRNA may be inhibited through microRNAs such as miR-203^36^. This suggests that PSMD14 inhibition can suppress both growth and metastasis through SMAD3 induction and SLUG reduction.

Although we identified PSMD14 as a molecular target for melanoma, there is currently no available drug targeting PSMD14. As the disturbance of proteasome function by PSMD14 knockdown may affect melanoma growth, proteasome inhibitors, including bortezomib, may be attractive drugs for melanoma. Bortezomib was approved by the Food and Drug Administration (FDA) for multiple myeloma or mantle cell lymphoma, which targets PSMB5 in 20S core particles. As PSMD14 is in 19S regulatory particles, it may be another molecular target in proteasomes. Thus, a PSMD14 inhibitor can overcome acquired resistance to bortezomib, caused by the mutation of PSMB5^37^. As such, a PSMD14 inhibitor may be applicable not only to melanoma, but also to other cancers, including multiple myeloma. Recently, capzimin was identified as a chemical inhibitor specific to PSMD14^38^. Therefore, drugs targeting PSMD14 can inhibit melanoma growth and metastasis.

## Supplementary information

Supplementary Information.
