# Supplementary material for: Targeting PSMD14 inhibits melanoma growth through SMAD3 stabilization
Source: Sci Rep. 2020 Nov 5;10:19214. doi: 10.1038/s41598-020-76373-y (PMC7644625; doi:10.1038/s41598-020-76373-y)
Supplement: Supplementary file 1 — Supplementary Information. [file 41598_2020_76373_MOESM1_ESM.pdf]

Supplementary information to:

Targeting PSMD14 inhibits melanoma growth through SMAD3 stabilization

Satoru Yokoyama[1,2 *], Yusuke Iwakami[2], Zhao Hang[2], Ryoei Kin[2], Yue Zhou[1], Yutaka Yasuta[1], Atsushi Takahashi[1], Yoshihiro Hayakawa[2], Hiroaki Sakurai[1]

[1]Department of Cancer Cell Biology, Faculty of Pharmaceutical Sciences; [2]Division of Pathogenic Biochemistry, Institute of Natural Medicine, University of Toyama, 2630 Sugitani, Toyama 930-0194, Japan.

Content:

Supplemental Figure 1

Supplemental Figure 2

Supplemental Figure 3

Supplemental Figure 1

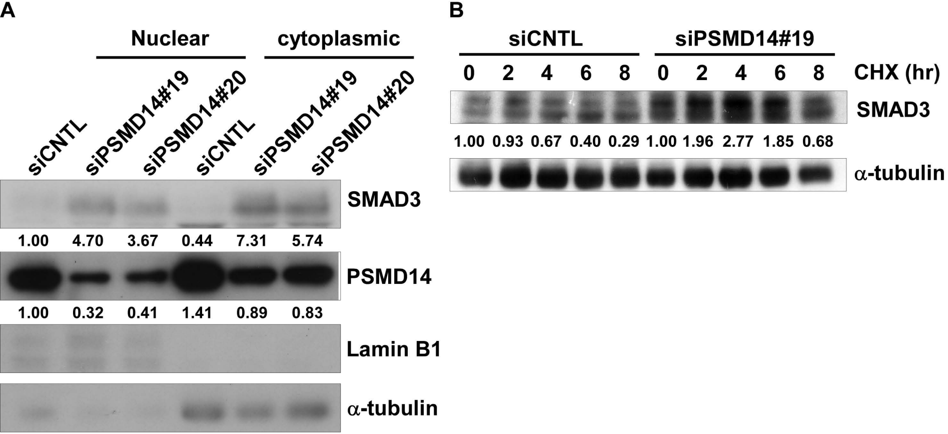

Supplemental Figure 1. PSMD14 regulates SMAD3 stability in M14 cells.
(A) M14 cells were transfected with PSMD14 siRNA for 96 hours. Nuclear or cytoplasmic protein was subjected to Western blotting. Other conditions were as in Figure 2C. (B) M14 cells were transfected with PSMD14 siRNA for 96 hours. The transfected cells were treated with 50 μg/ml of cycloheximide for the indicated times and subjected to the Western blotting. The band intensities were measured by ImageJ, normalized to that at 0 hours for each cell line, and shown below each panel.

Supplemental Figure 2

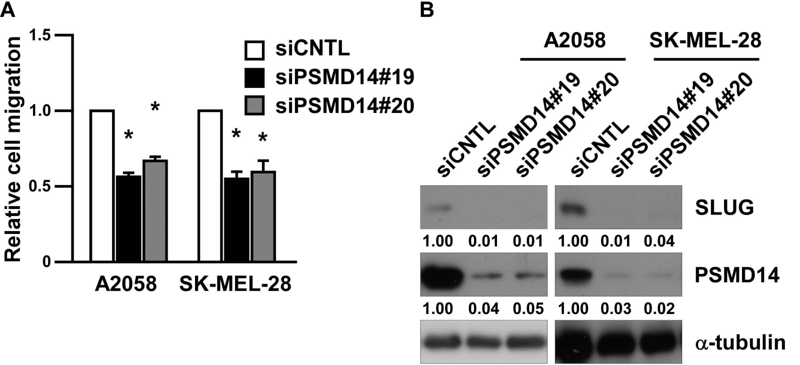

Supplemental Figure 2. Targeting PSMD14 inhibits melanoma migration and SLUG expression. (A) A2058 and SK-MEL-28 cells were transfected with PSMD14 siRNA for 96 hours and then subjected to the migration assay. *P<0.01 vs siCNTL-transfected cells by one-way ANOVA followed by the Bonferroni post hoc test. (B) A2058 and SK-MEL-28 cells were transfected with PSMD14 siRNA for 96 hours. The whole cell lysates were subjected to Western blotting.

Supplemental Figure 3

A

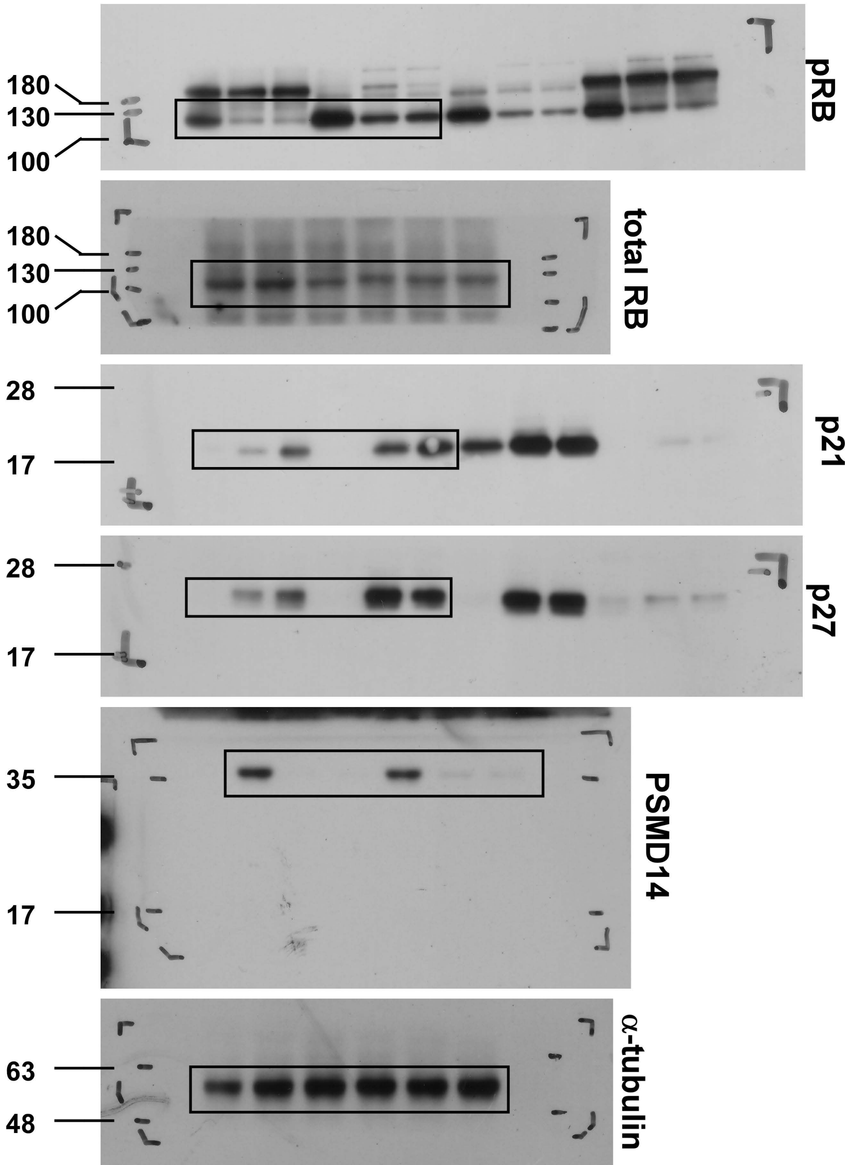

Supplemental Figure 3

B

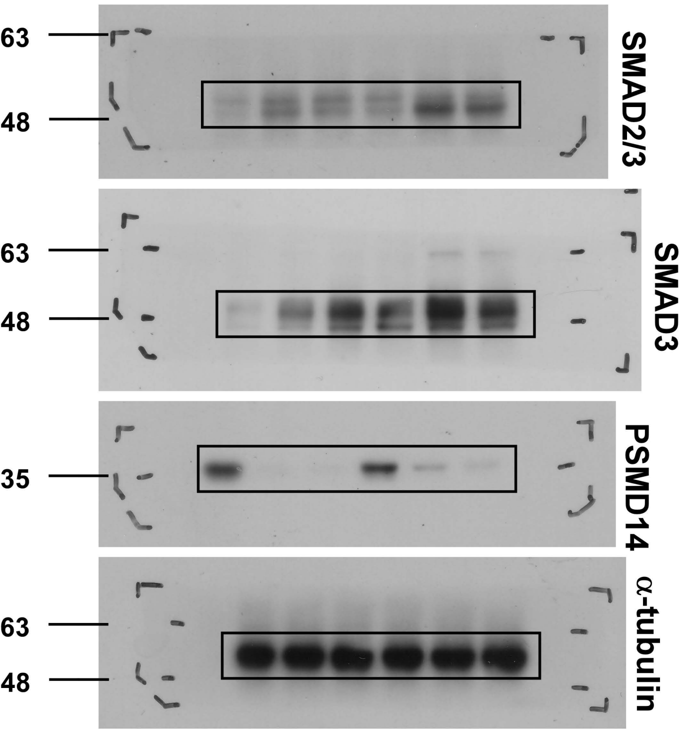

C

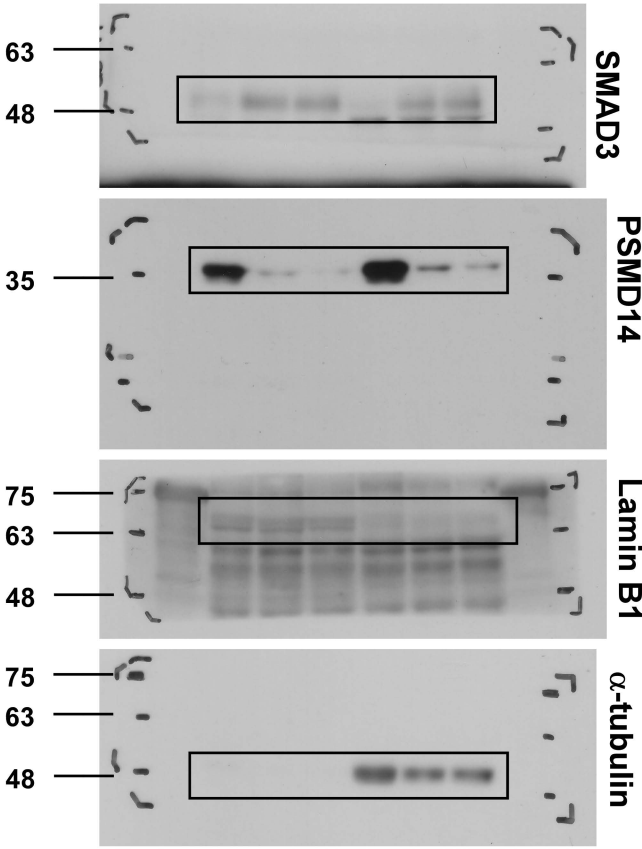

D

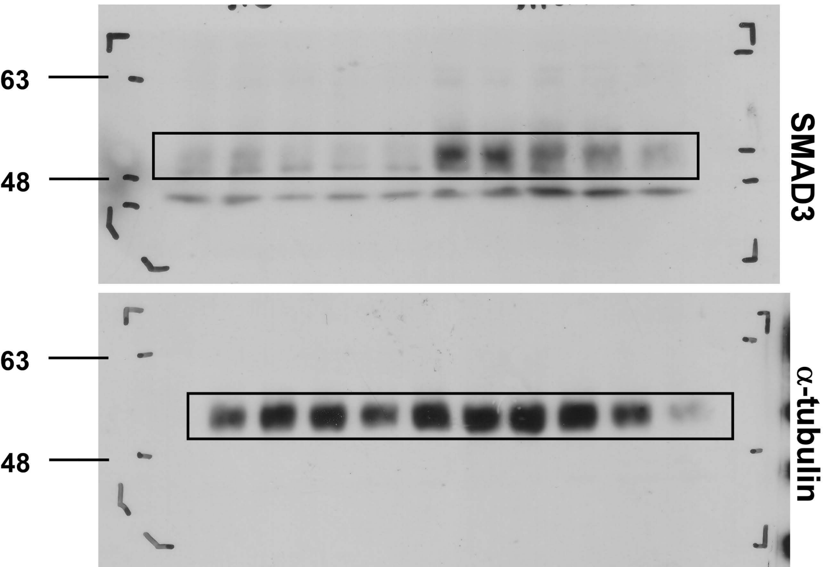

Supplemental Figure 3

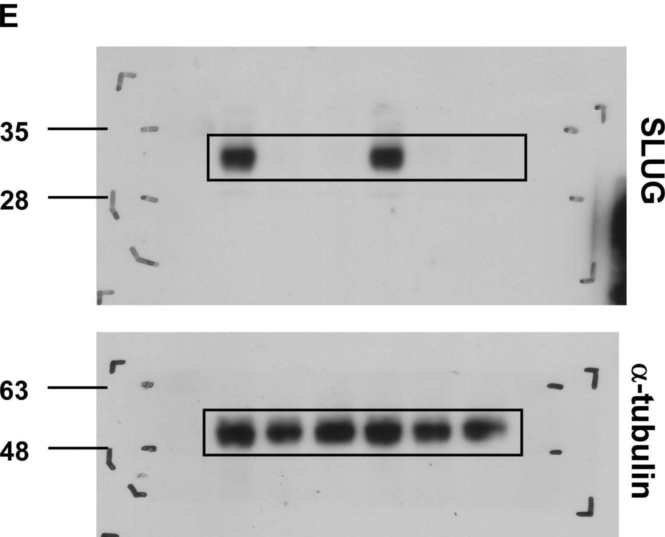

Supplemental Figure 3

F

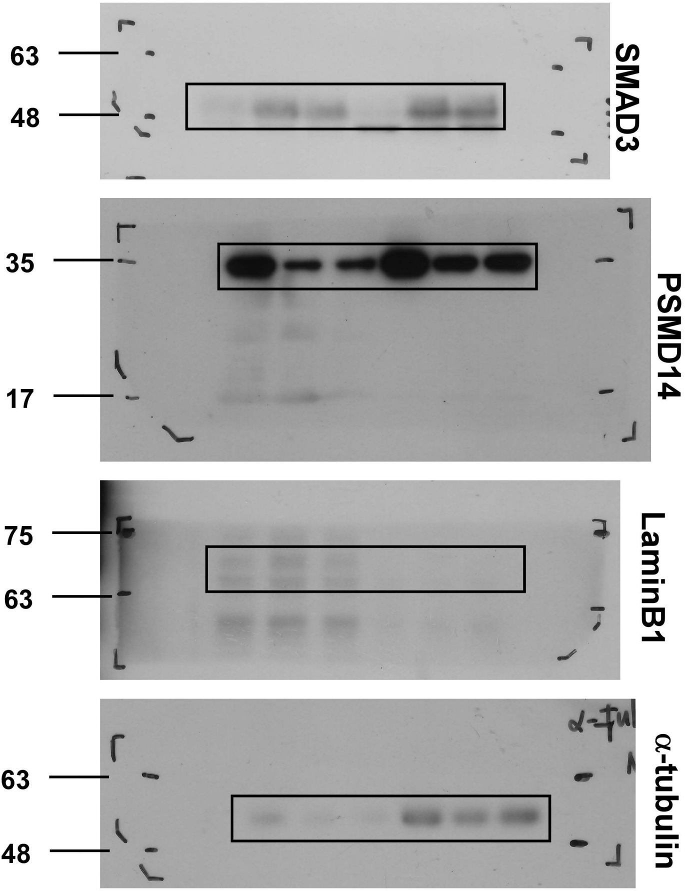

G

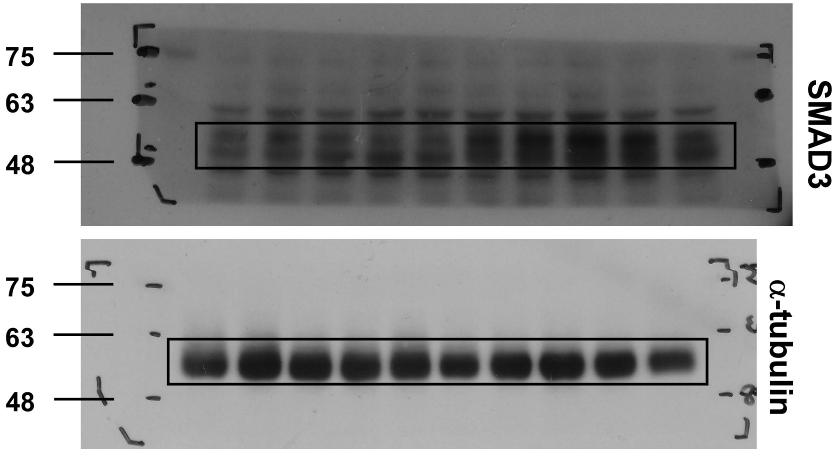

Supplemental Figure 3

H

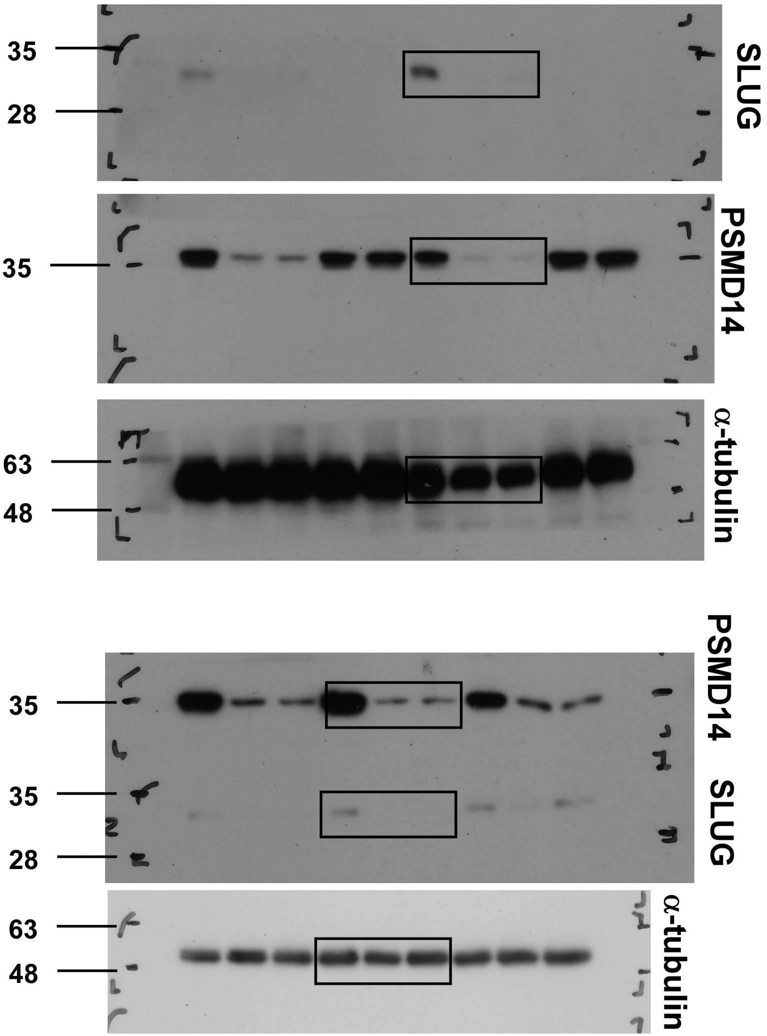

Supplemental Figure 3. Uncropped scans of all blots.
Uncropped scans of Figure 1D (A), Figure 2B (B), Figure 2C (C), Figure 2D (D), Figure 3B (E), Supplemental Figure 1A (F), Supplemental Figure 1B (G), and Supplemental Figure 2B (H) were shown.
